# The Effects of the COVID-19 Pandemic on Mental Health: A Web-Based Study Among Romanian Adults

**DOI:** 10.7759/cureus.31331

**Published:** 2022-11-10

**Authors:** Elena Popa, Teodora Tetia, Mihaela Poroch, Monica Ungureanu, Adriana Cosmescu, Liliana Barbacariu, Ana Maria Slanina, Agnes Bacusca, Antoneta Petroae, Otilia Novac, Mihaela Manole, Dana Anton-Paduraru, Andrei Emilian Popa, Elena-Adorata Coman

**Affiliations:** 1 Preventive Medicine, “Grigore T. Popa” University of Medicine and Pharmacy, Iași, ROU; 2 Preventive Medicine, "Grigore T. Popa" University of Medicine and Pharmacy, Iași, ROU; 3 Pediatrics, “Grigore T. Popa” University of Medicine and Pharmacy, Iași, ROU

**Keywords:** family medicine, covid-19, survey, depression, anxiety

## Abstract

Background

In Romania, as in other parts of the world, the family doctor is the first to make contact with a healthy patient and is also the first to notice even the smallest pathological changes. In the context of the severe acute respiratory syndrome coronavirus 2 (SARS-CoV-2) pandemic, the patient's communication with the family doctor became even closer and some behavioral changes could be easily noticed.

Objective

To assess the symptoms of anxiety and depression in the Romanian population using social media platforms in the context of the COVID-19 pandemic.

Methods

We conducted an anonymous, web-based cross-sectional survey consisting of 31 questions related to general characteristics (age, gender, education, inhabitancy, residence, smoking status, and alcohol consumption) and adapted GAD-7 (7-item General Anxiety Disorders questionnaire) and PHQ-9 (9-item Patient Health Questionnaire). This questionnaire was sent to volunteers in an electronic format through a social network (Facebook, Twitter). The data collected were statistically processed using IBM SPSS v25.0 (IBM Corp., Armonk, NY). The inclusion criteria were age over 18 years and no history of chronic disease. The exclusion criteria consisted of the absence of a mental illness diagnosis.

Results

From the 1254 respondents, 1232 cases were selected for statistical analysis after applying the exclusion criteria. The mean age was 35.94 (SD = 11.4, 95%CI=10.9-11.9) with a minimum of 18 years and a maximum of 97 years. Eighty-four point nine percent (84.9%; N=1046) of all study participants are female and 79.13% (N= 975) live in the urban area. A total of 188 (15.25%) were diagnosed with COVID-19 of which 31 (16.66%) were male and 157 (15%) were female. N=170 (13.8%) reported moderate symptoms of anxiety during the last two weeks before the survey while N=96 (7.8%) had severe anxiety. Twenty-two point two percent (22.2%; N=274) of the participants reported moderate symptoms of depression while 10.1% (N=125) had moderately severe symptoms and 6.6% (N=81) could be diagnosed with severe depression. A greater likelihood of screening for depression diagnosis was associated with ages between 25 and 34 years (OR=0.90, 95%CI=0.86-0.94, *P<.*001), 35 and 44 years (OR=0.88, 95%CI=0.84-0.93, *P<.*001), and 45 and 54 years (OR=0.87, 95%CI=0.82-0.92, *P<.*001). Also, a tendency was observed for women to be more prone to high levels of anxiety (OR=1.21, 95%CI=1.08-1.35, *P< .*001) and depression (OR=2.16, 95%CI=1.51-308, *P< .*005).

Conclusions

Regarding the high prevalence of depression and anxiety, especially in women, appropriate measures for the risk categories should be applied. In the new social context created by the COVID-19 pandemic, screening for psychiatric and psychological disorders should be performed by telemedicine.

## Introduction

In March 2020, the WHO declared the novel coronavirus (SARS-CoV-2) infection a pandemic. Along with the increased pressure on the medical system, the spread of the SARS-CoV-2 infection has generated multiple economic and social effects on society. The fear of being infected and the measures of social distancing (reducing social contact, limiting recreation) imposed to limit the transmission of the virus, along with financial instability generated by job loss, are the main causes of mental illness during the coronavirus disease 2019 (COVID-19) pandemic [1-3]. In this context, family physicians, along with other medical professionals, play an important role in managing psycho-emotional changes as part of the pandemic response [4]. In clinical practice during the pandemic, the monitoring of psychosocial needs and the support provided to patients during direct (face-to-face) meetings have been greatly reduced due to isolation/quarantine measures at home and due to restricted access to services caused by the rapid spread of SARS-CoV-2 virus infection. In this context, it is necessary to develop methods for the remote evaluation of patients by family doctors through telephone consultations and/or communication via the Internet [4].

In this study, we started with the hypothesis that in Romania the prevalence of anxiety and depression increased in the adult population during the COVID-19 pandemic.

This article was previously posted to the JMIR preprint server on December 30, 2020.

## Materials and methods

Participants and procedure

We conducted an anonymous, web-based cross-sectional survey on anxiety and depression among Romanian adult Internet users [5]. For our trial ISRCTN1465265, we developed a questionnaire that includes open and closed questions. The questionnaire had 31 questions regarding socio-demographic data, personal experience related to COVID-19 infection, and mental health status of which 16 were specific questions for the presence of anxiety or depression adapted from two well-recognized and validated tools: the GAD-7 (Generalized Anxiety Disorders-7) scale for diagnosing anxiety and the PHQ-9 scale (Nine-Item Patient Health Questionnaire) for diagnosing depression [6,7]. The questionnaire was sent to volunteers in an electronic format through a social network (Facebook, Twitter). Recruitment to the study was done on a voluntary basis, and participants completed a questionnaire available on a Google Drive link. Recruitment of subjects started on December 3, 2020, and finished on December 16, 2020. The questionnaire was made public on social media, and participants clicked on the link on Google Drive and answered questions only one time. Participants who were included in the study were healthy Romanian adult Internet users of both genders without any history of mental illness.

Ethical considerations

As this is a survey study, informed consent was inferred from the provision of information about participants at the beginning of the survey. All data collected were anonymous and confidential. The study was carried out in accordance with the updated Helsinki Declaration [8]. Participants were not remunerated and did not incur financial costs related to participation in the study.

Statistical analysis

Our online survey was reported according to the CHERRIES (Checklist for Reporting Results of Internet E-Surveys) list [9]. For data processing, we developed a statistical analysis plan. The data was collected in Microsoft Office Professional 2019, Excel (Microsoft Corporation, Redmond, WA), and was processed and analyzed using IBM SPSS v25.0 (IBM Corp., Armonk, NY) and its specific functions for descriptive and correlation analysis. Descriptive statistics were used to summarize the demographic variables. Pearson correlations were used to determine the correlations between the studied variables for all participants. Separate multivariate logistic regression analysis was performed to model associations of outcomes with demographic factors. The significance level was set at a p-value of < 0.05.

## Results

Sociodemographic characteristics

The survey was completed by 1254 participants, of which 22 subjects were excluded based on exclusion criteria (history of mental illness).

Hence, 1232 participants were included in the analysis. The mean age was 35.94 (SD = 11.4, 95%CI=10.9-11.9) with a minimum of 18 years and a maximum of 97 years. Eighty-four point nine percent (84.9%) of all study participants are female (N= 1046) and 79.13% live in an urban area (N= 975). Seventy-eight point thirty-eight percent (78.38%) of the participants were under 45 years old and 79.15% of the respondents had higher education (university and above; N=974) (Table 1).

**Table 1:** Sociodemographic characteristics of the study group

	Total (N=1232)	Men (N=186)	Women (N=1046)
The mean age (SD)	35. 94	37. 32	35. 70
	(SD = 11.4, 95%CI=10.9,11.9)	(SD=11.86, 95%CI=10.35,12.91)	(SD = 11.36 95%CI=10.84,12.03)
Age distribution (years)	N (%)	N (%)	N (%)
18-24	225 (18.26)	34 (18.27)	191 (18.26)
25-34	369 (29.95)	44 (23.65)	325 (31.07)
35-44	372 (30.19)	62 (33.33)	310 (29.63)
45-54	196 (15.9)	33(17.74)	163 (15.58)
55-64	47 (3.81)	9 (4.83)	38 (3.63)
> 65	23 (1.86)	4 (2.15)	19 (1.86)
Residency (%)			
Urban area	975 (79.31)	162 (87.96)	813 (77.72)
Rural area	257 (20.86)	24 (12.90)	233 (22.27)
Education (%)			
Primary	4 (0.32)	1(0.53)	3 (0,28)
Secondary	5 (0.40)	1 (0.53)	4 (0.38)
High school	249 (20.21)	37 (19.89)	212 (20.26)
University degree	676 (54.97)	104 (55.91)	572 (54.68)
Postgraduates	298 (24.18)	43 (23.11)	255 (24.37)

Twenty-seven point four percent (27.4%; N=338) of the respondents are smokers, 62.9% (N=775) drink alcohol at parties or special occasions while 3.6%(N= 44) have daily consumption but have less than three drinks, and 0.5% (N=6) respondents have more than three drinks daily.

One hundred eighty-eight (188, 15.25%) of the participants were diagnosed with COVID-19 before completing the questionnaire. Twelve point eight percent (12.8%; N=158) was quarantined as close contacts of a person who tested positive for the SARS-CoV-2 infection.

Descriptive statistics were used to summarize demographic variables and to characterize, on one side, the group as a whole and, on the other side, the subgroups determined by the COVID-19 status. A specific period of time before SARS-CoV-2 infection or the establishment of the isolation/quarantine measure was not used as an exclusion criterion. The frequency of participants for each subgroup was evaluated based on the presence of SARS-CoV2 infection as follows (Table 2):

**Table 2:** Distribution of participants in subgroups

	Gender						
	Men N (%)	Female N (%)	Chi-square (df)		Total N (%)		
Subgroup I (no diagnosis COVID-19/not quarantined as close contact)	131 (70.43)	755 (72.17)	0.352 (2), P > .05		886 (71.19)		
Subgroup II (quarantined as close contact)	24 (12.9)	134 (12.81)	0.346 (2), P > .05		158 (12.82)		
Subgroup III (diagnosed with COVID-19 and isolated)	31 (16.66)	157 (15.0)	0.332 (1), P > .05		188 (15.25)		
Total	186	1046			1232		

Subgroup I (no diagnosis of COVID-19/not quarantined as close contact) - 886 subjects; Subgroup II (quarantined as close contact with COVID-19 case) - 158 subjects; Subgroup III (diagnosed with COVID-19 and isolated) - 188 subjects.

Evaluation outcomes

Depression

The survey was built using adapted questions from the 9-item Patient Health Questionnaire (PHQ-9) to assess the extent of depressive symptoms in an otherwise healthy population. The scores varied from 0 to 27 points and a cut-off score for the diagnosis of depression was considered above 10 points, varying from moderate to severe (moderate - 10 to 14 points, moderately severe - 15 to 19 points, and severe - 20 to 27 points).

A percentage of 22.2% (N=274) of the participants reported moderate symptoms of depression with a PHQ-9 score between 10 and 14 points while 10.1% (N=125) had moderately severe symptoms and 6.6% (N=81) could be diagnosed with severe depression (Table 3).

**Table 3:** Outcome measures - depression PHQ-9: 9-item Patient Health Questionnaire

Depression	Total N=1232	Subgroup I N=886	Subgroup II N=158	Subgroup III N=188
No significant symptoms (PHQ-9 score 0-4)				
	344 (27.92)	259 (29.23)	36 (22.78)	49 (26.06)
Mild (PHQ-9 score 5-9)				
	408 (33.11)	282 (31.82)	59 (37.34)	67 (35.63)
Moderate (PHQ-9 score 10-14)				
	274 (19.48)	201 (22.68)	29 (18.35)	44 (23.40)
Moderately severe (PHQ-9 score 15-19)				
	125 (10.14)	83 (9.36)	21 (13.29)	21 (11.17)
Severe (PHQ-9 score > 20)				
	81 (6.57)	61 (6.88)	13 (8.22)	7 (3.72)

Anxiety

The assessment of anxiety symptoms was made by an adapted subset of questions from the 7-item Generalized Anxiety Disorders questionnaire (GAD-7). A cut-off score higher than 10 points was considered suggestive of anxiety symptoms. N=170 (13.8%) reported moderate symptoms of anxiety during the last two weeks before the survey while N=96 (7.8%) had severe anxiety with a GAD-7 score between 16 and 21 points (Table 4).

**Table 4:** Outcome measures- anxiety

	Total N=1232	Subgroup I N=886	Subgroup II N=158	Subgroup III N=188
No significant symptoms (GAD-7 score 0-5)				
	461 (37.41)	330 (37.24)	60 (37.97)	71 (37.76)
Mild (GAD-7 score 6-10)				
	505 (40.99)	365 (41.19)	55 (34.81)	85 (45.21)
Moderate (GAD-7 score 11 -15)				
	170 (13.79)	119 (13.41)	27 (17.08)	24 (12.76)
Severe (GAD-7 score 16-21)				
	96 (7.79)	72 (8.12)	16 (10.12)	8 (4.25)

There was no significant statistical correlation on the Pearson chi-square test between the diagnosis of COVID-19 and the levels of depression (R=10.568, df=3, P=.227) (Table 5) or anxiety (R=7.566, df=3, P-value=.56) (Table 6).

**Table 5:** Correlations between the COVID-19 diagnosis and the levels of anxiety

	Anxiety						
	No symptoms N (%)	Mild N (%)	Moderate N (%)	Severe N (%)	Chi-square(df)	Total N (%)	
Subgroup I (no diagnosis COVID-19/not quarantined as close contact)	330 (37.2)	365 (41.2)	119 (13.4)	72 (8.1)	26.724, 3, P> .001	886 (71.19)	
Subgroup II (quarantined as close contact)	60 (3.0)	55 (34.8)	27( 17.1)	16 (10.1)	12.222, 3, P > .001	158 (12.82)	
Subgroup III (diagnosed with COVID-19 and isolated)	71 (37.8)	85 (45.2)	24 (12.8)	8 (4.3)	7.566, 3, P> .001	188 (15.25)	

**Table 6:** Correlations between the COVID-19 diagnosis and the levels of depression

	Depression						
	No symptoms N (%)	Mild N (%)	Moderate N (%)	Moderately severe N(%)	Severe N(%)	Chi-square (df)	Total N(%)
Subgroup I (no diagnosis COVID-19/not quarantined as close contact)	259 (29.2)	282 (31.8)	201 (22.7)	83 (9.4)	61 (6.9)	25.328, 4, P> .001	886 (71.19)
Subgroup II (quarantined as close contact)	36 (22.8)	59 (37.3)	29 (18.4)	21 (13.3)	13 (8.2)	11.908, 4; P> .001	158 (12.82)
Subgroup III (diagnosed with COVID-19 and isolated)	49 (26.1)	67 (35.6)	44 (23.4)	21 (11.2)	7 (3.7)	10.486, 4; P > .001	188 (15.25)

In the subgroups studied, the frequency of anxiety levels varied according to gender as follows: N=27 (17.1%) of the women in the Subgroup II had moderate symptoms of anxiety while N=15 (9.5%) had severe symptoms compared to men who had no symptoms of moderate anxiety and only N=1 (0.6%) were affected by severe symptoms. The results of multivariate logistic regression analyses are presented in Table 7.

**Table 7:** Multivariate logistic regression analyses

	GAD-7 Score OR (95%CI)	PHQ-9 Score OR (95%CI)
Age group		
18-24 years	1.00 (ref.)	1.00 (ref.)
25-34 years	1.08* (1.02-1.12)	0.90*** (0.86-0.94)
35-44 years 45-54 years	1.04 (0.98-1.11) 1.01 (0.94-1.09)	0.88*** (0.84-0.93) 0.87*** (0.82-0.92)
55-64 years	0.98 (0.86-1.11)	0.87* (0.79-0.96)
Over 65 years	0.95 (0.81-1.12)	0.94 (0.83-1.06)
Gender		
Men	1.00 (ref.)	1.00 (ref.)
Women	1.21** (1.08-1.35)	2.16*** (1.51-3.08)
Education		
Primary education	1.00 (ref.)	1.00 (ref.)
University education	0.79 (0.59-1.04)	1.04 (0.83-1.30)
High school	0.80 (0.60-1.06)	1.04 (0.83-1.30)
Secondary education	0.70 (0.45-1.05)	1.18 (0.86-1.61)
Postgraduate education	0.79 (0.59-1.06)	1.01 (0.80-1.27)
Smoking status non-smoker	1.00(ref.)	1.00 (ref.)
Smoker	1.01 (0.97-1.06)	1.03 (0.99-1.06)
Inhabitancy		
Urban area	1.00 (ref.)	1.00 (ref.)
Rural area	1.01 (0.96-1.06)	1.008 (0.97-1.04)
Residence		
Apartment	1.00 (ref.)	1.00 (ref.)
House	0.98 (0.94-1.02)	1.01 (0.98-1.05)
Covid-19 status		
No diagnosis	1.00 (ref.)	1.00 (ref.)
Quarantined as contact	1.02 (0.98-1.07)	0.99 (0.93-1.05)
Diagnosed and isolated	1.04* (1.01-1.09)	0.92* (0.87-0.98)

In our group, a greater likelihood of screening for depression diagnosis was associated with ages between 25 and 34 years (OR=0.90, 95%CI=0.86-0.94, P<.001), 35-44 years (OR=0.88, 95%CI=0.84-0.93, P<.001), and 45-54 years (OR=0.87; 95%CI=0.82-0.92, P<.001). Also, a tendency was observed for women to be more prone to high levels of anxiety (OR=1.21, 95%CI=1.08-1.35, P<.001) and depression (OR=2.16, 95%CI=1.51-3.08, P< .005).

Studying the correlations between variables, we found a strong positive correlation between the calculated values of the GAP-7 and PHQ-9 scores (r=.812; P< .001) (Table 8).

**Table 8:** Correlations between GAP-7 and PHQ-9 scores GAP-7: 7-item General Anxiety Disorders questionnaire; PHQ-9: 9-item Patient Health Questionnaire

		GAD-7 score	PHQ-9 score
GAD-7 score	Pearson Correlation	1	.812^**^
	Sig. (2-tailed)		.000
	N	1232	1232
PHQ-9 score	Pearson Correlation	.812^**^	1
	Sig. (2-tailed)	.000	
	N	1232	1232

## Discussion

During the last year, we have noticed a change in attitude among the adult population, either in face-to-face consultations or in teleconsultations. By the nature of the specialty, the family doctor ends up noticing even a minor change in the general mood of her/his patients. Although noted by the physician, patients are reluctant to declare symptoms probably because of the stigma that a diagnosis of mental illness has. The idea of ​​the online questionnaire strengthened our theory that electronic anonymity leads to a surplus of openness.

The objective of our anonymous, cross-sectional, web-based survey was to assess the impact of the COVID-19 pandemic on mental health among the adult population using social networks. We mention that it is the first study in Romania of this type completed at this time. We focused on two mental health conditions during the COVID-19 pandemic: depression and anxiety.

We used social networks for the voluntary recruitment of patients for two reasons: 1. in Romania more than half of the adults use social networks (11, 34 million social network users in 2020) [10]; 2. subjects often prefer an anonymous online interview about mental health issues to “face-to-face” approach.

Regarding our study group, the majority of the respondents were women (84.9%), which can be interpreted as a higher availability of online communication for Romanian women as compared to men. In addition, we also noticed that most subjects live in urban areas, which can be explained by lower internet resources, but also by a higher percentage of functional illiteracy in rural areas [11].

Furthermore, in our opinion, the difficulties of the population with low educational status, in reading, understanding, and completing the questionnaire led to a larger number of participants with higher education and can be considered a limitation of our study. An extensive on-site study could bring new discussions about mental illness in this population category.

Principal results

Our main findings revealed the high prevalence of anxiety and depression in people who responded to this questionnaire distributed through social networks.

Depression is one of the main mental disorders, with an impact at the individual level by decreasing quality of life [2] and collectively by increasing pressure on the health system [12]. As a consequence of the COVID-19 pandemic, experts estimated an increase in anxiety and depression in all geographical areas.

The prevalence of depression during the pandemic varies depending on the geographical area and the population studied but also on the method (scale) used to diagnose these conditions [1,3-4,13-17]. Thus, in the meta-analysis of 66 studies (N = 221. 970) published by Wu et al. [13], the general prevalence of depression and anxiety was 31.4% and 31.9%, respectively, while Cenat et al. [1] in their meta-analysis of 55 studies (N = 189.159) show much lower values (15.97% for depression, respectively, 15.15% for anxiety).

In our country, the preliminary results of the international study COH-FIT (collaborative outcome study on health and functioning during infection times) showed that the coronavirus pandemic affected the mental health of the Romanian population. Thus, 42% of respondents reported a deterioration in stress, a symptom that was present at a higher level in women (46%) and young adults (47%) [18]. In our study, we reported that more than one-third of respondents (38.9%; N = 480) experienced depression during the pandemic period.

The prevalence calculated by us in this online survey is much higher than that reported for our country (6%) in 2017, in WHO statistics for the general population [19]. We note that in post-communist Romania, there was an increase in the number of people with specific symptoms of depression in the ‘90s, an increase triggered by the lack of adaptation to the new capitalist system, which affected adults aged between 40 and 55 years [20].

Depression is the most common mental health problem for women [19]. Compared to men, women predominate in three conditions: depression, anxiety, and somatic complaints [21]. In the pandemic period, consistent with existing data, which also notes that depression may be more persistent in females [21], in our study, we reported that women are more affected by depression compared to men.

Although anxiety disorders are considered less serious psychiatric disorders [2], the impact on quality of life and the impossibility of normal professional activity draw attention to this mental health problem. The prevalence of anxiety in the pandemic period varies depending on the geographical area and the population studied but also on the scale used to diagnose anxiety [3-4].

In our study, for the pandemic period, moderate and severe anxiety (GAD-7 score >10) was experienced by 21.5% of participants, higher than the prevalence of anxiety (3.7%) previously reported for Romania before the pandemic [19]. Women reported anxiety more frequently than men did (Table 7), similar to existing data on mental health during the pandemic [22-25]. Seventeen point one percent (17.1%) of the women quarantined after close contact with a patient diagnosed with COVID-19, had moderate symptoms of anxiety while 9.5% had severe symptoms compared to men who had no symptoms of moderate anxiety and only 0.6% were affected by severe symptoms.

Interestingly, the highest percentage of anxiety was recorded in quarantined subjects as direct contacts with a confirmed case of COVID-19 and not in patients confirmed with COVID-19. We can link this finding to the characteristics of the studied participants. For example, Subgroup III (with a history confirmed by COVID-19) includes subjects with a mean of 36.4 ± 11.23 years and no associated chronic disease and who, according to current medical evidence [22- 23], are not included in the risk categories for the development of a complicated form of SARS-COV-2 infection.

Using statistical analysis, we found a strong positive correlation between the two diagnostic scores, GAD-7 and PHQ-9. This correlation is important because depression and anxiety often occur together, especially in family medicine practice [24-25]. Increased recognition of depression and anxiety comorbidity disorders is important because it will lead to more effective treatment of these conditions.

However, given the increased prevalence of anxiety and depression in the population studied by us, at the level of the public health system, it is necessary to adopt programs aimed at the population vulnerable to stress induced by the COVID-19 pandemic.

Future perspective

Digital healthcare technologies can be used to provide easier, faster, and more cost-effective access to mental health care [26]. An application can be introduced in the electronic work program of the family doctor, through which patients can regularly fill in questionnaires regarding their mental health status, the results of which will be transmitted and reported quickly.

Limitations

The present study is based on a one-time assessment, which limits the possibility of distinguishing between an acute and a chronic psychiatric condition. Although respondents who stated that they had been diagnosed with a mental illness were excluded, the patient's medical history cannot be verified, which is why there may be psychiatric patients included in the study group.

As described above [27], the disadvantage of voluntary web surveys is that the conclusions are not based on a representative sample and, therefore, cannot be extrapolated to the entire population. Due to online recruitment, young users of social networks and highly educated participants are seen more frequently in these surveys.

## Conclusions

Although we expected a difference in the levels of anxiety and depression based on the reported symptoms between the subgroups determined by the COVID-19 status, the study revealed an increased prevalence of anxiety and depression compared to other reports made in our country, especially in women since they are already a social category at risk. For this reason, the family physician should pay closer attention during teleconsultations and face-to-face consultations in the current epidemiological context when addressability for specialized medical services is lower.

## References

[1] Cénat JM, Blais-Rochette C, Kokou-Kpolou CK (2021). Prevalence of symptoms of depression, anxiety, insomnia, posttraumatic stress disorder, and psychological distress among populations affected by the COVID-19 pandemic: A systematic review and meta-analysis. Psychiatry Res.

[2] Nguyen HC, Nguyen MH, Do BN (2020). People with suspected COVID-19 symptoms were more likely depressed and had lower health-related quality of life: the potential benefit of health literacy. J Clin Med.

[3] Pfefferbaum B, North CS (2020). Mental health and the Covid-19 pandemic. N Engl J Med.

[4] Gray DP, Freeman G, Johns C, Roland M (2020). Covid 19: a fork in the road for general practice. BMJ.

[5] Wang X, Cheng Z (2020). Cross-sectional studies: strengths, weaknesses, and recommendations. Chest.

[6] Spitzer RL, Kroenke K, Williams JB, Löwe B (2006). A brief measure for assessing generalized anxiety disorder: the GAD-7. Arch Intern Med.

[7] Löwe B, Kroenke K, Herzog W, Gräfe K (2004 ). Measuring depression outcome with a brief self-report instrument: sensitivity to change of the Patient Health Questionnaire (PHQ-9). J Affect Disord.

[8] (2020). WMA Declaration of Helsinki - ethical principles for medical research involving human subjects. https://www.wma.net/policies-post/wma-declaration-of-helsinki-ethical-principles-for-medical-research-involving-human-subjects/.

[9] Eysenbach G (2012). Correction: improving the quality of web surveys: the checklist for reporting results of Internet e-surveys (CHERRIES). J Med Internet Res.

[10] Sava JA (2020). Social media usage in Romania - statistics & facts. https://www.statista.com/topics/7134/social-media-usage-in-romania.

[11] Sturzoiu A, Balan M (2018). The functional illiteracy in the knowledge society: a case of Romania. I E Letters.

[12] Brahmbhatt A, Richardson L, Prajapati S (2021). Identifying and managing anxiety disorders in primary care. Prim Care Companion J Clin Psychiatry.

[13] Wu T, Jia X, Shi H, Niu J, Yin X, Xie J, Wang X (2021). Prevalence of mental health problems during the COVID-19 pandemic: a systematic review and meta-analysis. J Affect Disord.

[14] Rodríguez-Rey R, Garrido-Hernansaiz H, Collado S (2020). Psychological impact and associated factors during the initial stage of the coronavirus (COVID-19) pandemic among the general population in Spain. Front Psychol.

[15] Sherman AC, Williams ML, Amick BC, Hudson TJ, Messias EL (2020). Mental health outcomes associated with the COVID-19 pandemic: prevalence and risk factors in a southern US state. Psychiatry Res.

[16] Zajacova A, Jehn A, Stackhouse M, Choi KH, Denice P, Haan M, Ramos H (2020). Mental health and economic concerns from March to May during the COVID-19 pandemic in Canada: insights from an analysis of repeated cross-sectional surveys. SSM Popul Health.

[17] Every-Palmer S, Jenkins M, Gendall P (2020). Psychological distress, anxiety, family violence, suicidality, and wellbeing in New Zealand during the COVID-19 lockdown: a cross-sectional study. PLoS One.

[18] Marica I (2020). The emotional impact of COVID-19: Romanians are sadder and angrier, study shows. https://www.romania-insider.com/covid-romanians-sadder-angrier-study.

[19] (2017). WHO. Depression and other common mental disorders: global health estimates. http://pp..

[20] Ionescu I (2005). Depression in post-communist Romania. Lancet.

[21] (2022). WHO. Mental health. https://www.who.int/health-topics/mental-health#tab=tab_1.

[22] Salzberger B, Buder F, Lampl B (2020). SARS-CoV-2/Covid-19 - Epidemiologie und Prävention [Article in German]. Nephrologe.

[23] (2020). Assessing risk factors for severe COVID-19 illness. https://www.cdc.gov/coronavirus/2019-ncov/covid-data/investigations-discovery/assessing-risk-factors.html.

[24] Groen RN, Ryan O, Wigman JTW (2020). Comorbidity between depression and anxiety: assessing the role of bridge mental states in dynamic psychological networks. BMC Med.

[25] McCarron RM, Shapiro B, Rawles J, Luo J (2021). Depression. Ann Intern Med.

[26] Folker MP, Helverskov T, Nielsen AS, Jørgensen US, Larsen JT (2018). Telepsychiatry provides new opportunities for the prevention and treatment of mental health disorders [Article in Danish]. Ugeskr Laeger.

[27] de Pedrazza P, Guzi M, Tijders K (2020). Life dissatisfaction and anxiety in COVID-19 pandemic. Luxembourg : European Union.

